# Hierarchical Flax Fibers by ZnO Electroless Deposition: Tailoring the Natural Fibers/Synthetic Matrix Interphase in Composites

**DOI:** 10.3390/nano12162765

**Published:** 2022-08-12

**Authors:** Nicoleta Preda, Andreea Costas, Francesca Sbardella, Maria Carolina Seghini, Fabienne Touchard, Laurence Chocinski-Arnault, Jacopo Tirillò, Fabrizio Sarasini

**Affiliations:** 1 National Institute of Materials Physics, Atomistilor 405A, 077125 Magurele, Romania; 2 Department of Chemical Engineering Materials Environment, Sapienza-Università di Roma & UdR INSTM, Via Eudossiana 18, 00184 Roma, Italy; 3 Département Physique et Mécanique des Matériaux, ENSMA, Institut PPRIME, CNRS—ENSMA—Université de Poitiers, 1 Av. Clément Ader, B.P. 40109, CEDEX, 86961 Poitiers, France

**Keywords:** composites, flax fibers, ZnO nanostructures, interface/interphase, electroless deposition

## Abstract

Hierarchical functionalization of flax fibers with ZnO nanostructures was achieved by electroless deposition to improve the interfacial adhesion between the natural fibers and synthetic matrix in composite materials. The structural, morphological, thermal and wetting properties of the pristine and ZnO-coated flax fibers were investigated. Thus, the ZnO-coated flax fabric discloses an apparent contact angle of ~140° immediately after the placement of a water droplet on its surface. An assessment of the interfacial adhesion at the yarn scale was also carried out on the flax yarns coated with ZnO nanostructures. Thus, after the ZnO functionalization process, no significant degradation of the tensile properties of the flax yarns occurs. Furthermore, the single yarn fragmentation tests revealed a notable increase in the interfacial adhesion with an epoxy matrix, reductions of 36% and 9% in debonding and critical length values being measured compared to those of the pristine flax yarns, respectively. The analysis of the fracture morphology by scanning electron microscopy and X-ray microtomography highlighted the positive role of ZnO nanostructures in restraining debonding phenomena at the flax fibers/epoxy resin matrix interphase.

## 1. Introduction

In the last two decades, environmental and economic concerns have been the engine in the development of composites based on natural fibers [1,2,3]. Derived from renewable resources, natural fibers offer significant biodegradability and sustainability, their composites being widely utilized in components for various industries, due to their low environmental impact. Thus, natural fiber-reinforced epoxy resins are the most used composite materials in the automotive sector (epoxy resin reinforced with flax, hemp, kenaf or sisal fibers for automotive components) and construction sector (furniture, decks, building materials—masonry, cementitious materials, thermal insulating materials, etc.). In this type of application, the mechanical properties of natural fibers-reinforced epoxy resin composites are essential, the interphase between the fibers and matrix playing the key role in achieving the required mechanical performance.

The main advantages of natural fibers are their specific features, namely high abundance, availability, renewability, biodegradability, recyclability, lightweight, low cost, ease of handling, good strength, very good thermal insulation and non-toxicity. The natural plant fibers such as flax, hemp, jute, etc. are actually cellulose-based materials being mainly made from cellulose, lignin, hemicellulose, pectin and wax [4], the percentage of each chemical component in the composition depends on the type of the fibers. Accordingly, due to a large number of hydroxyl groups from the cellulose structure, the natural fibers present an inherent hydrophilicity nature that is responsible for the limited adhesion and the poor compatibility between these fibers and polymer matrices (e.g., epoxy resin, polypropylene) used in the composite materials which usually feature a hydrophobic behavior [5]. One of the most used approaches for modifying the wettability of natural fibers in order to improve the interfacial bonding and enhance the properties of the composite materials consists in the physical or chemical treatment of the fiber surface [5,6]. The combination between natural fibers and inorganic nanostructures can also lead to an improvement in the mechanical performance of the composites [7], expanding their potential range of applications. Therefore, recently, the surface functionalization of various natural plant fibers (flax [8,9,10,11,12], sisal [13], fique [14], hemp [15] and cotton [16,17]) with metal oxide nanostructures (TiO_2_ [9,11,12], ZnO [8,10,13,14,15,16,17]) has received considerable attention [18]. One of the main aims is to mimic naturally occurring biological systems, such as wood or nacre, which rely on the development of structures at different length scales (from the macro to the nanoscale) for achieving interesting combinations of strength and toughness [19]. Among the cellulose-based materials, flax fibers are extensively used as reinforcement in composite materials due to their high specific mechanical properties [20,21,22], their functionalization with ZnO or TiO_2_ nanostructures by solution-based methods such as hydrothermal [8], sol-gel [11], in situ chemical synthesis [10] or dip-coating [9,12] being recently reported.

Abundant and low-cost material, ZnO is an interesting choice for the functionalization of natural fibers being one of the most investigated metal oxides. Furthermore, ZnO can be easily prepared in nanostructures with different morphologies by simple and scalable wet and dry methods [23,24,25,26]. Usually, the chemical synthesis of ZnO by solution-based approaches involves inexpensive equipment, easily accessible and low-cost chemical reagents, mild temperature, and ambient pressure facilitating the large-scale production and the application of the materials and devices based on this metal oxide. Consequently, ZnO is a technological key material which can be applied in superhydrophobic surfaces [15,16,24,25], sensing [25], field effect transistors [23], photodetectors [27], photovoltaic cells [28], etc. Among the solution-based techniques, the ZnO electroless deposition is a successful surface functionalization technique that allows the coverage of complex shapes and non-conductive substrates such as natural (cotton, hemp) or synthetic (polyester, polyamide, poly(lactic acid)) fabrics, these studies being mainly focused on the wetting properties of the ZnO-functionalized fabrics [15,16]. It must be noticed that in the electroless process, before the immersion of the substrate in the deposition solution containing a zinc salt and a reducing agent, its surface must contain catalytic metal sites because the ZnO growth takes place only on the metal-catalyzed surface being triggered by the increase of the pH in the vicinity of this surface via the redox reactions [15,16]. Recently, this functionalization approach has proven to be effective also on basalt fibers (natural fabric of mineral origin), and the presence of ZnO nanostructures on the surface of these fibers had a positive effect on their mechanical performance [29]. Thus, due to a relatively uniform coating on large areas and good adhesion of the ZnO nanostructures to the fiber surface, the improvement in the apparent interfacial shear strength of the ZnO-coated basalt fibers by electroless deposition was higher in comparison with that obtained for the basalt fibers decorated with ZnO nanorods by a hydrothermal treatment, where a poor chemical bonding between the metal oxides nanostructures and the surface of the fibers was observed [30].

In this context, the aim of this study is to obtain hierarchical flax fibers by ZnO electroless deposition, this wet chemical approach offering the possibility to modify the surface of the flax fibers with ZnO nanostructures for enhancing the natural fibers/synthetic matrix interfacial adhesion. Although ZnO electroless deposition can be regarded as a scalable functionalization technique, very suitable for achieving a high degree of functionalization of flax fibers, which further can be integrated into composites with enhanced mechanical performance, up to now there are no reports on the coating of the flax fibers by ZnO electroless deposition. Till now, from natural fibers of cellulosic origin, only cotton and hemp fibers were coated by ZnO electroless deposition, the studies revealing the UV-blocking and superhydrophobic properties of the ZnO-functionalized fabrics [15,16]. Additionally, only two papers were focused on the mechanical properties of flax fibers functionalized with ZnO nanostructures through a hydrothermal treatment, the growth process involving a ZnO seed layer previously deposited on the fiber surface [8,31]. Moreover, natural fibers of mineral origin such as basalt fibers were lately coated by ZnO electroless deposition, the work emphasizing a significant improvement in the apparent interfacial shear strength (~42%) with limited degradation of the pristine basalt fiber tensile strength (a reduction of ~17%) [29]. Thus, ZnO nanostructured interphase obtained on the surface of flax fibers by electroless deposition can allow a smoother transition between the flax yarns and the epoxy resin matrix in terms of mechanical properties for a better load transfer and mechanical interlocking. In particular, structural and morphological properties of the pristine and functionalized flax fabrics were investigated. A thermal and wettability analysis of the fabrics was also performed before and after the functionalization, while to characterize the interfacial adhesion, tensile tests were carried out on yarns and on single-yarn composite samples allowing the determination of the influence of the ZnO electroless deposition. Consequently, the new information provided by this research regarding the design of ZnO nanostructured interphase on flax fibers can be very helpful for developing natural fiber-reinforced epoxy resin composites with enhanced mechanical performance that can find potential applications in various industries, especially in automotive and construction fields.

## 2. Materials and Methods

### 2.1. Materials

In the ZnO electroless deposition were used unsized flax fabric samples supplied by Composites Evolution (UK) with a 2 × 2 twill architecture and a fiber areal weight of 200 g/m^2^. In addition, single flax yarns were manually extracted from the flax fabrics for assessing the influence of the sample geometry (fabric or yarn) on the growth of ZnO nanostructures. Single yarn fragmentation tests were carried out for evaluating the interfacial adhesion of the functionalized flax yarns. In this case, an epoxy matrix supplied by Gurit, commercially known as PRIME 27, was mixed with a PRIME 20 slow hardener in a recommended 100:28 weight ratio, the curing cycle being 7 h at 90 °C.

### 2.2. ZnO Electroless Deposition

The chemical reagents (SnCl_2_, PdCl_2_, Zn(NO_3_)_2_ and (CH_3_)_2_NHBH_3_) were purchased from Sigma Aldrich (Burlington, VT, USA). The functionalization of flax fabrics or flax yarns by ZnO electroless deposition included three steps: sensitization, activation and deposition, the optimal reaction parameters being chosen based on our previous study carried out on cotton, another cellulosic fiber [16]. At first the substrates were immersed at room temperature 2 h in the sensitization aqueous solution (40 g/L SnCl_2_ and 20 mL/L HCl (37% vol.)) followed by 2 h in the activation aqueous solution (0.1 g/L PdCl_2_ and 20 mL/L HCl (37% vol.)). Further, the substrates with metal-catalyzed surfaces were immersed for 2 h, at 70 °C, in the deposition aqueous solution containing 0.07 M Zn(NO_3_)_2_ and 0.01 M (CH_3_)_2_NHBH_3_. The obtained samples were carefully rinsed with distilled water and dried under the ambient atmosphere.

**Figure 1:**
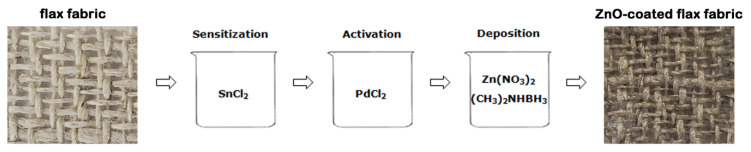
Schematic representation of the steps involved in the functionalization of the flax fabrics by ZnO electroless deposition.

A schematic representation of the functionalization of flax fabrics by ZnO electroless deposition is depicted in Figure 1.

### 2.3. Structural, Morphological, Thermal and Wettability Analysis of the Pristine and Functionalized Flax Fabrics

The structure, morphology and elemental composition of the samples were investigated by X-ray diffraction (XRD), field emission scanning electron microscopy (FESEM) and energy dispersive X-ray analysis (EDX) using a Bruker AXS D8 Advance instrument with Cu Kα radiation (λ = 0.154 nm), a Zeiss Merlin Compact field emission scanning electron microscope and a Zeiss Gemini SEM 500 field emission scanning electron microscope equipped with energy dispersive X-ray analysis Quantax Bruker XFlash detector 610 M as an accessory, respectively. The wetting properties were evaluated using a Drop Shape Analysis System, model DSA100 from Kruss GmbH by recording with a video camera the modification of the volume of a water droplet placed on the surface of the sample. The thermal degradation behavior of the modified flax fabrics was investigated by thermogravimetric analysis (TGA), the tests being carried out with a SetSys Evolution TGA analyzer (Setaram Instrumentation). Thus, the samples weighing about 25–30 mg were placed in an alumina pan and subjected to a heating rate of 10 °C/min in a temperature range of 25–800 °C, in a nitrogen atmosphere.

### 2.4. Characterization of the ZnO-Coated Flax Yarns: Tensile Test and Single Yarn Fragmentation Test

The effect induced by the presence of the ZnO nanostructures on the surface of the flax yarns on their mechanical properties was evaluated, the tensile tests being carried out on modified single flax yarns. Prior to the measurements, the samples were conditioned for 24 h at 45 °C. Further, the tensile tests were performed according to ASTM C-1557 with three different gauge lengths, namely 20, 30, and 40 mm [8] using a Zwick/Roell Z010 equipped with a 1 kN load cell in displacement control (crosshead speed = 2 mm/min). For each gauge length, the results are the average of thirty measurements.

The level of the interfacial adhesion between modified flax yarns and epoxy matrix was investigated by single yarn fragmentation tests. Thus, the well-known single fiber fragmentation test was adapted to test single yarns, the details of this test and its assumptions being reported in previous works [8,32,33,34]. Specimens containing one single flax yarn were tested in tension with a crosshead speed of 0.005 mm/min by using an Instron E1000 ElectroPuls test machine equipped with a load cell of 2 kN. The yarn diameters and fragment lengths were evaluated by optical microscopy using a ZEISS Axio Imager optical microscope. It has to be mentioned that the application of tensile loading was interrupted in the case of specimen failure or when the fragmentation saturation level was reached, meaning that no new yarn breaks appeared during a subsequent strain increase by 0.5%. The results stem from at least 10 valid fragmentation tests. Numerical data were statistically analyzed by ANOVA and *t*-test by using an alpha value of 0.05 using Origin software.

After single yarn fragmentation tests, the fracture morphology of the single yarn composites was investigated by a Tescan Mira3 field emission scanning electron microscope. Prior to FESEM analysis, the samples were sputter coated with a gold thin layer. Fractured samples were also subjected to X-ray microtomography (micro-CT) analysis using an UltraTom CT scanner by RX Solutions with a resolution of 1.5 μm, an accelerating voltage of 50 kV and a beam current of 157 μA. An Avizo 9.0 software was used for the volumetric reconstruction of the X-ray images acquired from 1120 rotation views over 360° (approximately 0.32° rotation step).

## 3. Results and Discussion

**Figure 2:**
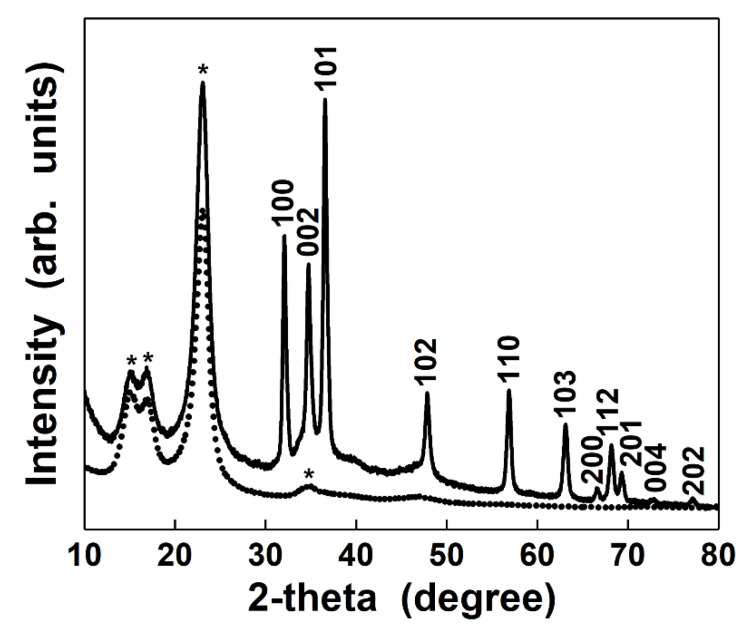
XRD patterns of flax (dotted curve) and ZnO-coated flax fibers (solid curve) as fabrics. * Peaks attributed only to flax fibers.

The XRD patterns of the flax fabrics before and after ZnO electroless deposition are presented in Figure 2. Both samples showed two narrow peaks at 15° and 17° and one sharper intense peak at 23° all related to the cellulose I phase from higher plant cellulose [35,36,37]. The deposition of ZnO on the surface of the cellulose-based fabrics is confirmed by the diffraction peaks assigned to its hexagonal wurtzite phase (JCPDS file no. 36-1451), the sharp and narrow diffraction peaks proving a well-crystallized material.

**Figure 3:**
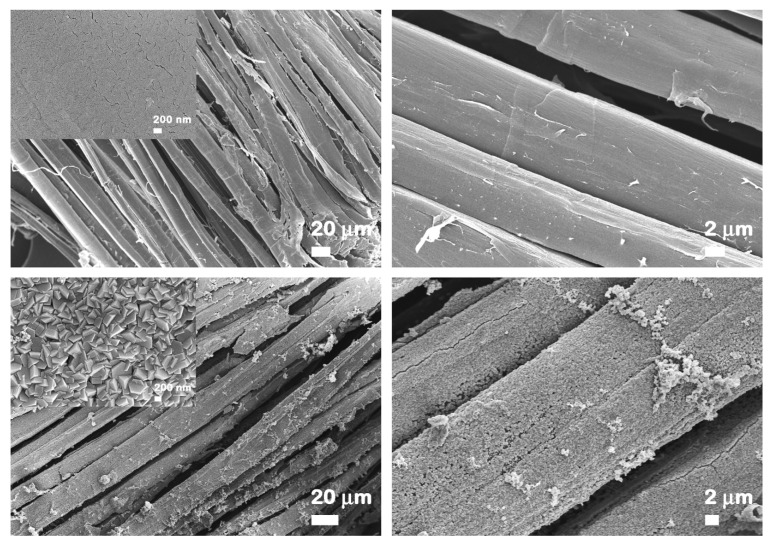
FESEM images at different magnifications of flax fibers (**top**) and ZnO-coated flax fibers (**bottom**) as fabrics.

**Figure 4:**
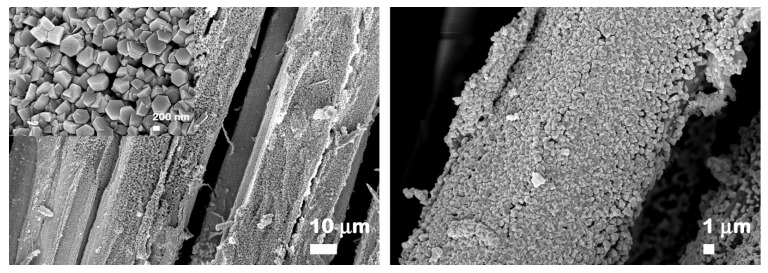
FESEM images at different magnifications of ZnO-coated flax fibers as yarns.

FESEM micrographs at different magnifications displaying the morphology of the flax fabrics before and after ZnO functionalization are included in Figure 3. The flax fabrics are formed by fibers with a relatively smooth surface having diameters in the range from 5 µm to 30 µm. After the ZnO electroless deposition, the surface of the natural fibers is covered by a continuous, homogeneous and densely packed array of ZnO well faceted hexagonal prisms having a base diameter of ~500 nm and the height of ~100 nm. The FESEM images of the flax yarns shown in Figure 4 reveal the same hexagonal prism shape for the ZnO, the structures having similar sizes to those deposited on flax fabrics. At higher magnifications (insets from Figure 3 and Figure 4), the FESEM images evidence that the crystallites are in fact formed by twin hexagonal prisms, oriented and connected along the c-axis of the growth direction, this being the characteristic morphology of the ZnO nanostructures deposited by electroless deposition on various natural or synthetic fibers [15,16].

At lower magnification, the FESEM images disclose that: (i) the most uniformly covered fibers are the outermost ones (Figure 3 and Figure 4) and (ii) the aggregates of ZnO particles are formed above the compact deposited layer (Figure 3).

These results can be explained considering the key role played by the diffusion processes involved in each of the three steps, an effect which is also supported by the inherent hydrophilicity nature of the pristine flax fabrics. In the sensitization step, the hydrolysis of SnCl_2_ generates Sn^2+^ ions that are adsorbed on the fibers’ surface through an electrostatic attraction. Further, in the activation step, the presence of PdCl_2_ triggered the oxidation of Sn^2+^ ions into Sn^4+^ ions and simultaneously Pd^2+^ ions are reduced to metallic Pd forming Pd^0^ colloids on the fibers’ surface. Finally, in the deposition step, the Zn(NO_3_)_2_ is hydrolyzed and the (CH_3_)_2_NHBH_3_ is oxidized in the presence of Pd catalyst releasing free electrons (its oxidation is well explained in Ref. [38]), which reduces NO_3_^−^ ions to NO_2_^−^ ions inducing at the same time an increase of HO^−^ ions concentration in the local area. The HO^−^ ions combine with the Zn^2+^ ions leading to the formation of Zn(OH)_2_ which forms ZnO by spontaneous dehydration.

All the mentioned chemical reactions are controlled by diffusion; therefore, they will take place faster where diffusion is promoted, i.e., on the outermost layer of the fibers, and much slower on the inner fibers of the textile material where diffusion is hampered. The catalytic reaction stimulates the nucleation of the ZnO crystallites from the Pd catalyst and, as the reactions continue, their growth continues covering completely the metal-catalyzed surface. The presence of the ZnO aggregates can be the result of a reaction that takes place in the volume of the deposition solution due to a local increase in the pH but which is still limited to the proximity of the functionalized surface. It is worth mentioning that no precipitate was observed in the volume of the solution or later decanted on the bottom of the deposition vessel.

**Figure 5:**
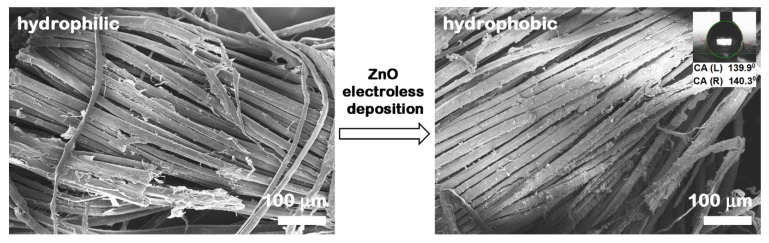
FESEM images of flax and ZnO-coated flax fibers as fabrics. In the inset is shown the optical photograph of the water droplet shape on the hydrophobic surface of ZnO-coated flax fabrics and the water contact angles for the corresponding sample.

**Figure 6:**
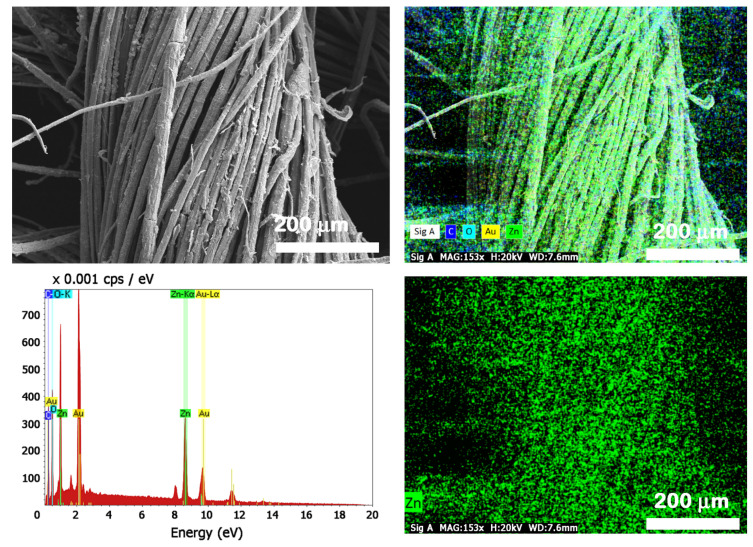
FESEM image, EDX mapping and EDX spectrum of ZnO-coated flax fabrics and the EDX mapping only for Zn element.

The FESEM micrographs taken on the large area presented in Figure 5 prove that the electroless deposition is a suitable functionalization route that allows to maintain a large specific surface area. It can be seen that the flax fibers were covered by a continuous nanostructured film formed by ZnO crystallites without embedding the flax fibers into a metal oxide thick layer. Furthermore, the distribution of Zn on the fabric surface and its atomic percentage in the sample were evaluated from the EDX mapping and EDX spectrum provided in Figure 6, the EDX data being acquired on areas having ~740 × 553 µm (width × height) in different locations of the flax fabrics. Thus, the EDX mapping image illustrates a uniform distribution of Zn on the surface of the flax fibers, the Zn atomic percentage being estimated at ~9–13% depending on the investigated area.

Further, the wettability of the flax fabrics before and after ZnO electroless deposition was evaluated (Figure 5). The pristine flax fabric absorbs instantly the water droplet placed on its surface (hydrophilic behavior). In the case of ZnO-coated flax fabrics, although immediately after the placement on the surface, the water droplet seems “to rest” on the fabric surface revealing an apparent contact angle of ~140° (a value that suggests a hydrophobic behavior). This particular behavior can be related on one hand to the decrease of the space between the flax fibers due to their covering with ZnO nanostructures and to the hydrophobic behavior of the ZnO synthesized by the electroless deposition approach [16] and on the other hand to the capillary wicking, a phenomenon that usually occurs in the porous structure of the fabrics [39]. It has to be mentioned that various studies were recently performed on flax fibers and their composites manufactured by liquid composite molding processes for understanding the capillary wicking phenomena, and the wetting parameters such as static and dynamic contact angles, polar and dispersive components of surface-free energy, and the swelling effects at various fiber scale (individual fiber, yarn, tow or fabric) [40,41,42,43,44].

**Figure 7:**
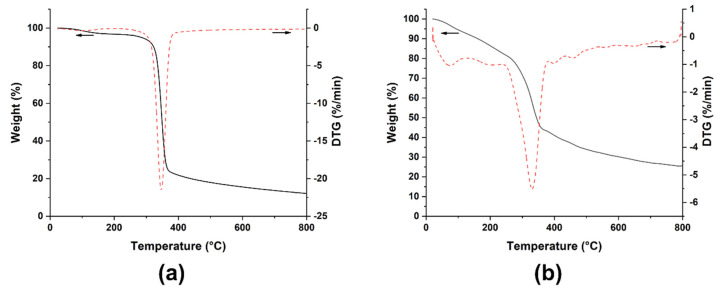
TGA (black solid curves) and dTG (red dot curves) thermograms of (**a**) neat flax yarns and (**b**) ZnO-coated flax yarns (arrows indicate the corresponding Y axis).

The thermal stability of the ZnO-coated flax yarns was evaluated by TGA analysis, the results shown in Figure 7 revealing a similar degradation behavior with that recorded for the pristine flax fibers [8]. The thermogram shows the three typical degradation steps for the cellulose-based materials: (i) around 80–100 °C, a first mass loss related to the moisture removal from the amorphous regions of cellulose, (ii) around 200–250 °C, a second peak linked to the decomposition of hemicellulose, the less thermally stable constituent of the lignocellulosic fibers and (iii) around 300–350 °C, a third peak due to the cellulose degradation; moreover, around 400–450 °C, a small tail related to the lignin degradation can be observed [45]. Consequently, compared to the pristine flax fibers (Figure 7a) [8], the global degradation behavior of the flax fibers is not significantly influenced by the ZnO nanostructures deposited on the fibers’ surface, albeit a decrease in the onset of thermal degradation was noted, likely ascribed to the premature decomposition of residues pertaining to the sensitization step. In fact, the presence of ceramic nanostructures can interfere with the thermal conductivity of the fibers and the resulting decrease might delay the hemicellulose/cellulose degradation [11]. This is further confirmed by the possible heat shielding effect of ZnO nanostructures, which should improve the thermal stability of the fibers [13,46]. The results confirm that the effect of nanostructures is dependent on the specific synthesis technique used.

**Figure 8:**
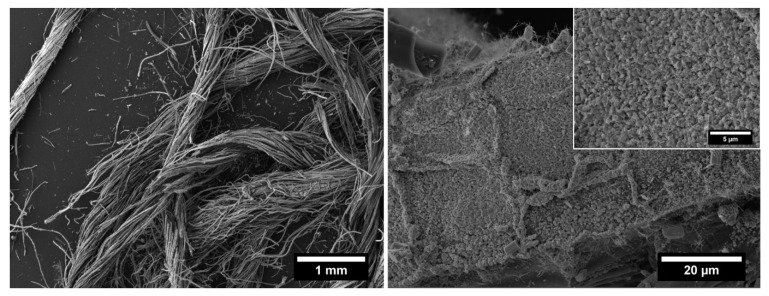
FESEM images at different magnifications of ZnO-coated flax yarns after the thermogravimetric test.

Further, the final residual mass obtained for ZnO-coated flax yarns (25.4%) compared to that recorded for neat flax yarns (13–17% [8]) indicates a high amount of ZnO deposited on flax fibers’ surface (~8–12%) that is thermally stable in the temperature range investigated [47]. The result is also confirmed by the FESEM analysis carried out on the modified flax fibers after the thermogravimetric test, i.e., after having experienced a temperature of 800 °C. The FESEM images shown in Figure 8 prove that well-individualized flax fibers are still uniformly covered by ZnO nanostructures.

Mechanical properties of the functionalized natural fibers are generally dependent on a number of parameters including the plant origin and growth conditions, fiber extraction method, fiber diameter variability, fiber surface treatment, moisture amount, etc., but also on the type of functionalization method applied for modifying the fibers’ surface. Therefore, the mechanical properties of flax yarns were investigated at different gauge lengths providing the following breaking forces at 20, 30 and 40 mm gauge lengths: 20.1 ± 3.4 N, 19.4 ± 2.9 N and 18.7 ± 3.1 N, respectively. Gauge length does not affect negatively the tensile properties of flax yarns, despite the higher probability of including a defect (e.g., kink bands) with increasing yarn length [48,49], as confirmed by ANOVA (*p* > 5%). Considering the comparison of mechanical properties of ZnO-coated flax yarns with those of neat flax yarns (22.9 ± 3.9 N) [8], both at a gauge length of 20 mm, it can be concluded that the ZnO growth by electroless deposition process does not compromise the tensile properties of underlying flax fibers, despite the corresponding properties are statistically different as confirmed by a *t*-test (*p* < 5%); this is in line with other studies dealing with flax fibers functionalized with nanostructures. Foruzanmehr et al. [11] reported on the importance of an oxidation pre-treatment to enhance the interfacial adhesion of flax fibers with a TiO_2_ coating by sol–gel dip-coating. The presence of TiO_2_ coating was supposed to increase the stress transfer among the fibrils and heal the fiber surface defects. The same reasoning was proposed by Ajith et al. [50] who decorated flax fibers with zirconia nanoparticles. The presence of hydrous zirconia nanoparticles reduced the surface defects, thus not degrading the mechanical properties.

**Figure 9:**
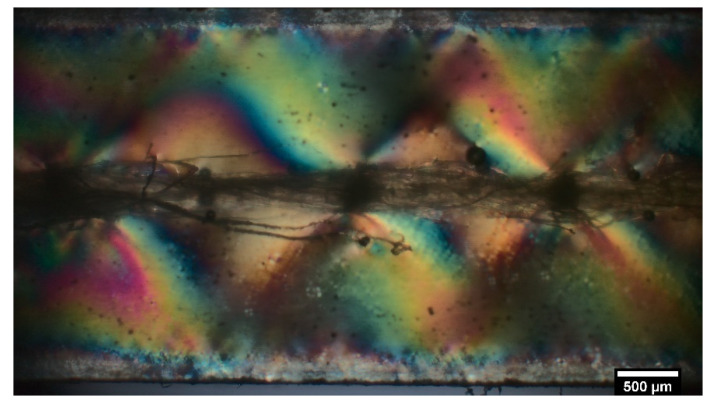
Birefringence effects of fragmented ZnO-coated flax yarns.

The effect of the ZnO nanostructures on the interfacial adhesion in flax/epoxy composites was evaluated by implementing the single yarn fragmentation test in accordance with the methodology reported in [33] and taking into account the limitations of this test when applied to the natural fibers (especially to natural fibers as yarns). In fact, the evaluation of the key parameter in this test, the interfacial shear strength (IFSS), is based on a homogenous distribution of the fiber diameters that is not the case for the natural fibers, these being characterized by a significant variability of fiber diameters among fibers and along the same fiber itself. Previous works have demonstrated that other parameters, such as the critical fragment length and debonding length provide a clear representation of the stress transfer efficiency at the yarn/matrix interface [32,33]. The critical fragment length (*l_c_*) was calculated according to Equation (1):

(1) $$l_{c}=\frac{4}{3}\bar{l}$$

where $\bar{l}$ is the average value of the fragment length. The yarn diameter and fragment lengths were measured in the gauge length zone by optical microscopy for each sample. In particular, the fragment lengths were determined as the average values between the internal and the central points of the breaking zones, which can be easily imaged by using polarized light. In this case, it is possible to observe the birefringence within the epoxy matrix, as shown in Figure 9. The dark area around each yarn breakpoint corresponds to the debonding length (*l_debonding_*) at the flax yarn/matrix interface [33]. The next step is the evaluation of the yarn strength at the critical length, *σ_f_* (*l_c_*), which cannot be calculated directly by experimental tests conducted at such short lengths. In this case, an extrapolation procedure was used from the knowledge of the distribution of the resistance values measured at greater lengths. In fact, in accordance with the analysis reported in [51], considering that the mean strength *σ_f_* can be obtained by Equation (2) (for a two-parameter Weibull distribution):

(2) $$\sigma_{f}=\sigma_{0}l^{-\frac{1}{m}}\Gamma \left(1+\frac{1}{m}\right)$$

and taking the logarithm of Equation (2), it can be written that:

(3) $$ln\sigma_{f}=-\frac{1}{m}lnl+ln\left[\sigma_{0}\Gamma \left(1+\frac{1}{m}\right)\right]$$

where *m* and *σ*_0_ are the shape and the scale parameters of the equivalent Weibull distribution, respectively, *l* the gauge length, *σ_f_* the tensile strength and Γ the Gamma function. A plot of *ln*(*σ_f_*) versus *ln*(*l*) should provide a linear trend with a slope equal to (−1/*m*). To this aim, in the present work, tensile tests were performed at three different gauge lengths, namely 20, 30, and 40 mm, obtaining a linear graph with the slope—−1/*m* = −0.135. The determination of the IFSS was addressed by using Equation (4):

(4) $$\tau =\frac{\sigma_{f}\left(l_{c}\right)d}{2l_{c}}$$

where *τ* is the interfacial shear strength, *σ_f_* (*l_c_*) is the yarn strength at the critical yarn length, *l_c_* is the critical fragment length and *d* is the yarn diameter. All the relevant parameters obtained from the fragmentation tests are summarized in Table 1, where values for the neat flax yarns [33] are also included as a reference.

From the results given in Table 1, the presence of ZnO nanostructures on the surface of flax fibers reduces both the critical and debonding length values of the flax yarns, these parameters being directly related to the extent of the fiber/matrix interfacial adhesion. Shorter fragments and a lower debonding length suggest that the stress transfer efficiency is improved, this effect occurring for strong interfacial bonds [52]. The difference in the debonding length resulted to be statistically significant as confirmed by a *t*-test (*p* < 5%). When considering the value of IFSS, an inconsistency can be found, as the value for the modified flax yarns is lower than the neat flax yarns; however, this is not completely surprising if one considers that this parameter is dependent on the yarn diameter (Equation (4)) and the natural fibers display a high variability in diameters. Furthermore, when adapting the fragmentation test to yarns, the cross-sectional area measurement of the yarn can include voids between the fibers that are then replaced by matrix during the impregnation step of the single yarn, without considering the yarn swelling occurred during the ZnO electroless deposition, which resulted in higher diameters (Table 1). An increase in average yarn diameter leads to a decrease in tensile strength, thus affecting the final IFSS values. All these considerations suggest that the numerical IFSS values can be considered only as qualitative ones.

**Figure 10:**
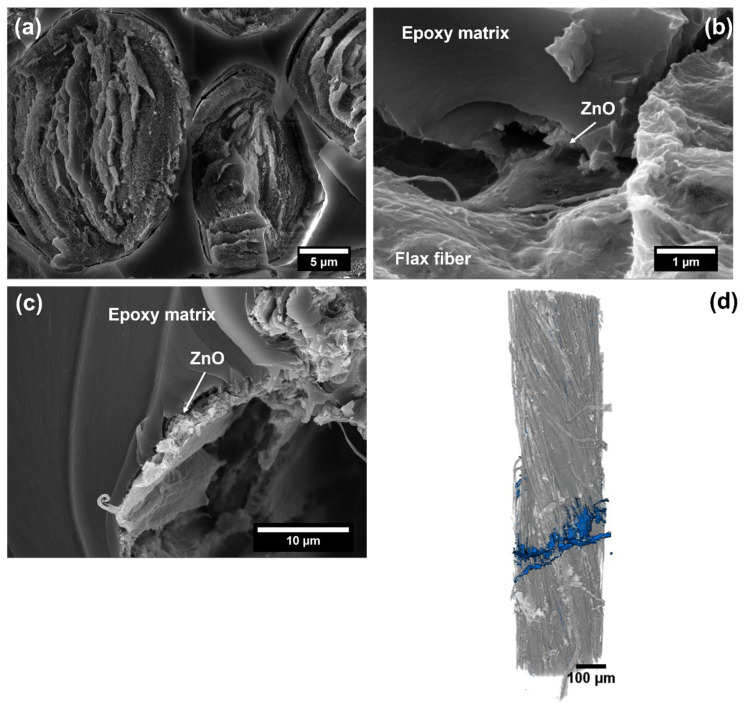
(**a**–**c**) FESEM images, at different magnifications, detailing the fracture morphology of a single ZnO-coated flax yarn composite after the single yarn fragmentation test and (**d**) volumetric reconstruction from micro-CT scans of ZnO-coated flax yarn (dark grey) and the fracture zone (blue).

The better interfacial adhesion was also supported by a morphological investigation performed by FESEM and micro-CT, the results being presented in Figure 10.

The FESEM images reveal that most flax fibers are broken at the same level and on the same fracture plane (Figure 10a), without extensive pull-out phenomena. In addition, when debonding occurred, it is possible to observe ZnO nanostructures acting as a bridge between the yarn and the epoxy matrix (Figure 10b). In a relatively few cases (Figure 10c), the failure at the yarn/ZnO interphase occurred, thus suggesting that a potential future improvement would be the enhancement of the bond between the ZnO nanostructures and the flax substrate. In Figure 10d, thanks to a segmentation of the raw images, it was possible to distinguish the flax yarn from the fracture zone (colored in blue for clarity); this blue zone includes decohesion and yarn break. It can be seen that this fracture zone has a very limited extension, thus supporting the small values observed for the debonding length measurements. The results prove that the interfacial adhesion between the natural fibers and the synthetic matrix is improved due to a mechanical interlocking effect played by the ZnO nanostructures deposited on the flax fibers by the electroless deposition approach. Indeed, the effects of grafting nanostructures on enhanced fiber/matrix interfacial adhesion are commonly ascribed to the interlocking interface created by nanostructures that bridge the matrix and the fibers [12] and to the higher strength and toughness of the nano-composite layer located at the interface [53]. The presence of densely packed ZnO nanostructures increases the specific surface area of the fibers, and the penetration of ZnO into the epoxy matrix improves further the mechanical interlocking; these effects have been demonstrated to be more important than the intrinsic properties of the individual constituents [13,54].

## 4. Conclusions

Electroless deposition, a scalable wet chemical functionalization approach, was successfully used to functionalize flax fibers with ZnO nanostructures. The grown ZnO displayed a hexagonal wurtzite structure with twin hexagonal prism morphology, and the flax fabric coated with ZnO nanostructures revealed an apparent contact angle of ~140°, immediately after the placement of a water droplet on its surface, further the water droplet being absorbed into the fabric. In addition, the electroless deposition preserved the tensile properties of the flax yarns without inducing significant changes in their global degradation behavior as determined by TGA analysis. The beneficial effect of this functionalization approach on the flax/epoxy interfacial adhesion was assessed by single yarn fragmentation tests. The significant reductions in the debonding and critical fragment length values obtained in the case of ZnO-coated flax yarns confirm the improvement of flax/matrix interphase providing new opportunities for tailoring the mechanical performance of the composites based on natural fibers and synthetic matrices. Another important feature of the proposed functionalization approach might consider the piezoelectric properties of the synthesized ZnO nanostructures that could be exploited in new multifunctional materials.

## Figures and Tables

**Table 1:** Diameter, critical length (*l_c_*), debonding length (*l_debonding_*) and interfacial shear strength values (IFSS) for ZnO-coated flax/epoxy single yarn composite and for neat flax/epoxy single yarn composite.

Specimen	Diameter (µm)	*l_c_* (µm)	*l_debonding_* (µm)	IFSS (MPa)
Neat flax/epoxy	248.33 ± 33.91	2687 ± 631	444 ± 49	19.3 ± 3.7
ZnO-coated flax/epoxy	398.87 ± 17.48	2452.7 ± 169.9	285.4 ± 84.4	14.7 ± 1.1

## Data Availability

Not applicable.

## References

[1] Li M., Pu Y., Thomas V.M., Yoo C.G., Ozcan S., Deng Y., Nelson K., Ragauskas A.J. (2020). Recent advancements of plant-based natural fiber–reinforced composites and their applications. Compos. Part B Eng..

[2] Thyavihalli Girijappa Y.G., Mavinkere Rangappa S., Parameswaranpillai J., Siengchin S. (2019). Natural fibers as sustainable and renewable resource for development of eco-friendly composites: A comprehensive review. Front. Mater..

[3] Bourmaud A., Beaugrand J., Shah D.U., Placet V., Baley C. (2018). Towards the design of high-performance plant fibre composites. Prog. Mater. Sci..

[4] Faruk O., Bledzki A.K., Fink H.-P., Sain M. (2012). Biocomposites reinforced with natural fibers: 2000–2010. Prog. Polym. Sci..

[5] Zhou Y., Fan M., Chen L. (2016). Interface and bonding mechanisms of plant fibre composites: An overview. Compos. Part B Eng..

[6] Kabir M.M., Wang H., Lau K.T., Cardona F. (2012). Chemical treatments on plant-based natural fibre reinforced polymer composites: An overview. Compos. Part B Eng..

[7] Hasan K.M.F., Horváth P.G., Alpár T. (2020). Potential Natural Fiber Polymeric Nanobiocomposites: A Review. Polymers.

[8] Sbardella F., Lilli M., Seghini M.C., Bavasso I., Touchard F., Chocinski-Arnault L., Rivilla I., Tirillo J., Sarasini F. (2021). Interface tailoring between flax yarns and epoxy matrix by ZnO nanorods. Compos. Part A Appl. Sci. Manuf..

[9] Prasad V., Sekar K., Varghese S., Joseph M.A. (2020). Evaluation of interlaminar fracture toughness and dynamic mechanical properties of nano TiO_2_ coated flax fibre epoxy composites. Polym. Test.

[10] Costa S., Ferreira D., Ferreira A., Vaz F., Fangueiro R. (2018). Multifunctional Flax Fibres Based on the Combined Effect of Silver and Zinc Oxide (Ag/ZnO) Nanostructures. Nanomaterials.

[11] Foruzanmehr M.R., Boulos L., Vuillaume P.Y., Elkoun S., Robert M. (2017). The effect of cellulose oxidation on interfacial bonding of nano-TiO_2_ coating to flax fibers. Cellulose.

[12] Wang H., Xian G., Li H. (2015). Grafting of nano-TiO_2_ onto flax fibers and the enhancement of the mechanical properties of the flax fiber and flax fiber/epoxy composite. Compos. Part A Appl. Sci. Manuf..

[13] Yang C., Han R., Nie M., Wang Q. (2020). Interfacial reinforcement mechanism in poly(lactic acid)/natural fiber biocomposites featuring ZnO nanowires at the interface. Mater. Des..

[14] Ovalle-Serrano S.A., Carrillo V.S., Blanco-Tirado C., Hinestroza J.P., Combariza M.Y. (2015). Controlled synthesis of ZnO particles on the surface of natural cellulosic fibers: Effect of concentration, heating and sonication. Cellulose.

[15] Frunza L., Preda N., Matei E., Frunza S., Ganea C.P., Vlaicu A.M., Diamandescu L., Dorogan A. (2013). Synthetic fabrics coated with zinc oxide nanoparticles by electroless deposition: Structural characterization and wetting properties. J. Polym. Sci. Part B Polym. Phys..

[16] Preda N., Enculescu M., Zgura I., Socol M., Matei E., Vasilache V., Enculescu I. (2013). Superhydrophobic properties of cotton fabrics functionalized with ZnO by electroless deposition. Mater. Chem. Phys..

[17] Wang X., Chen X., Cowling S., Wang L., Liu X. (2019). Polymer Brushes Tethered ZnO Crystal on Cotton Fiber and the Application on Durable and Washable UV Protective Clothing. Adv. Mater. Interfaces.

[18] Doineau E., Cathala B., Benezet J.-C., Bras J., Moigne N.L. (2021). Development of Bio-Inspired Hierarchical Fibres to Tailor the Fibre/Matrix Interphase in (Bio)composites. Polymers.

[19] Wang Y., Naleway S.E., Wang B. (2020). Biological and bioinspired materials: Structure leading to functional and mechanical performance. Bioact. Mater..

[20] Ramesh M. (2019). Flax (*Linum usitatissimum* L.) fibre reinforced polymer composite materials: A review on preparation, properties and prospects. Prog. Mater. Sci..

[21] Pickering K.L., Aruan Efendy M.G., Le T.M. (2016). A review of recent developments in natural fibre composites and their mechanical performance. Compos. Part A Appl. Sci. Manuf..

[22] Ku H., Wang H., Pattarachaiyakoop N., Trada M. (2011). A review on the tensile properties of natural fiber reinforced polymer composites. Compos. Part B Eng..

[23] Florica C., Costas A., Kuncser A., Preda N., Enculescu I. (2016). High performance FETs based on ZnO nanowires synthesized by low cost methods. Nanotechnology.

[24] Florica C., Preda N., Costas A., Zgura I., Enculescu I. (2016). ZnO nanowires grown directly on zinc foils by thermal oxidation in air: Wetting and water adhesion properties. Mater. Lett..

[25] Florica C., Preda N., Enculescu M., Zgura I., Socol M., Enculescu I. (2014). Superhydrophobic ZnO networks with high water adhesion. Nanoscale Res. Lett..

[26] Preda N., Enculescu M., Enculescu I. (2014). Polysaccharide-assisted crystallization of ZnO micro/nanostructures. Mater. Lett..

[27] Costas A., Florica C., Preda N., Apostol N., Kuncser A., Nitescu A., Enculescu I. (2019). Radial heterojunction based on single ZnO-Cu_x_O core-shell nanowire for photodetector applications. Sci. Rep..

[28] Socol M., Preda N., Costas A., Breazu C., Stanculescu A., Rasoga O., Popescu-Pelin G., Mihailescu A., Socol G. (2020). Hybrid organic-inorganic thin films based on zinc phthalocyanine and zinc oxide deposited by MAPLE. Appl. Surf. Sci..

[29] Preda N., Costas A., Lilli M., Sbardella F., Scheffler C., Tirillò J., Sarasini F. (2021). Functionalization of basalt fibers with ZnO nanostructures by electroless deposition for improving the interfacial adhesion of basalt fibers/epoxy resin composites. Compos. Part A Appl. Sci. Manuf..

[30] Lilli M., Sbardella F., Bavasso I., Bracciale M.P., Scheffler C., Rivilla I., Tirillò J., Xin W., De Rosa I.M., Sarasini F. (2020). Tailoring the interfacial strength of basalt fibres/epoxy composite with ZnO-nanorods. Compos. Interfaces.

[31] Sbardella F., Rivilla I., Bavasso I., Russo P., Vitiello L., Tirillò J., Sarasini F. (2021). Zinc oxide nanostructures and stearic acid as surface modifiers for flax fabrics in polylactic acid biocomposites. Int. J. Biol. Macromol..

[32] Seghini M.C., Touchard F., Sarasini F., Chocinski-Arnault L., Tirillò J., Bracciale M.P., Zvonek M., Cech V. (2020). Effects of oxygen and tetravinylsilane plasma treatments on mechanical and interfacial properties of flax yarns in thermoset matrix composites. Cellulose.

[33] Seghini M.C., Touchard F., Sarasini F., Chocinski-Arnault L., Mellier D., Tirillò J. (2018). Interfacial adhesion assessment in flax/epoxy and in flax/vinylester composites by single yarn fragmentation test: Correlation with micro-CT analysis. Compos. Part A Appl. Sci. Manuf..

[34] Seghini M.C., Touchard F., Chocinski-Arnault L., Placet V., François C., Plasseraud L., Bracciale M.P., Tirillò J., Sarasini F. (2020). Environmentally friendly surface modification treatment of flax fibers by supercritical carbon dioxide. Molecules.

[35] Moryganov A.P., Zavadskii A.E., Stokozenko V.G. (2018). Special Features of X-ray Analysis of Cellulose Crystallinity and Content in Flax Fibres. Fibre Chem..

[36] Mwaikambo L.Y., Ansell M.P. (2002). Chemical modification of hemp, sisal, jute, and kapok fibers by alkalization. J. Appl. Polym. Sci..

[37] French A.D. (2014). Idealized powder diffraction patterns for cellulose polymorphs. Cellulose.

[38] Van Den Meerakker J.E.A.M. (1981). On the mechanism of electroless plating. II. One mechanism for different reductants. J. Appl. Electrochem..

[39] Cotorobai V.F., Zgura I., Birzu M., Frunza S., Frunza L. (2016). Wicking behavior of fabrics described by simultaneous acquiring the images of the wet region and monitoring the liquid weight. Colloids Surf. A Physicochem. Eng. Asp..

[40] Vo H.N., Pucci M.F., Corn S., Le Moigne N., Garat W., Drapier S., Liotier P.J. (2020). Capillary wicking in bio-based reinforcements undergoing swelling–Dual scale consideration of porous medium. Compos. Part A Appl. Sci. Manuf..

[41] Liotier P.J., Pucci M.F., Le Duigou A., Kervoelen A., Tirilló J., Sarasini F., Drapier S. (2019). Role of interface formation versus fibres properties in the mechanical behaviour of bio-based composites manufactured by Liquid Composite Molding processes. Compos. Part B Eng..

[42] Pucci M.F., Liotier P.J., Seveno D., Fuentes C., Van Vuure A., Drapier S. (2017). Wetting and swelling property modifications of elementary flax fibres and their effects on the Liquid Composite Molding process. Compos. Part A Appl. Sci. Manuf..

[43] Pucci M.F., Liotier P.J., Drapier S. (2016). Capillary wicking in flax fabrics–Effects of swelling in water. Colloids Surf. A Physicochem. Eng. Asp..

[44] Pucci M.F., Liotier P.J., Drapier S. (2015). Capillary effects on flax fibers–Modification and characterization of the wetting dynamics. Compos. Part A Appl. Sci. Manuf..

[45] Yao F., Wu Q., Lei Y., Guo W., Xu Y. (2008). Thermal decomposition kinetics of natural fibers: Activation energy with dynamic thermogravimetric analysis. Polym. Degrad. Stab..

[46] Devi R.R., Maji T.K. (2012). Effect of Nano-ZnO on thermal, mechanical, UV stability, and other physical properties of wood polymer composites. Ind. Eng. Chem. Res..

[47] Chen W., Liu X., Liu Y., Kim H.-I. (2010). Synthesis of microcapsules with polystyrene/ZnO hybrid shell by Pickering emulsion polymerization. Colloid Polym. Sci..

[48] Hughes M. (2011). Defects in natural fibres: Their origin, characteristics and implications for natural fibre-reinforced composites. J. Mater. Sci..

[49] Baley C. (2002). Analysis of the flax fibres tensile behaviour and analysis of the tensile stiffness increase. Compos. Part A Appl. Sci. Manuf..

[50] Ajith A., Xian G., Li H., Sherief Z., Thomas S. (2016). Surface grafting of flax fibres with hydrous zirconia nanoparticles and the effects on the tensile and bonding properties. J. Compos. Mater..

[51] El Asloun M., Donnet J.B., Guilpain G., Nardin M., Schultz J. (1989). On the estimation of the tensile strength of carbon fibres at short lengths. J. Mater. Sci..

[52] Kim B.W., Nairn J.A. (2002). Observations of Fiber Fracture and Interfacial Debonding Phenomena Using the Fragmentation Test in Single Fiber Composites. J. Compos. Mater..

[53] Xian G., Walter R., Haupert F. (2004). Development of Tribologically Optimized Surface Coatings with Micro and Nano Particles. Materwiss Werksttech.

[54] Marriam I., Xu F., Tebyetekerwa M., Gao Y., Liu W., Liu X., Qiu Y. (2018). Synergistic effect of CNT films impregnated with CNT modified epoxy solution towards boosted interfacial bonding and functional properties of the composites. Compos. Part A Appl. Sci. Manuf..

